# Classification and Identification of Plant Fibrous Material with Different Species Using near Infrared Technique—A New Way to Approach Determining Biomass Properties Accurately within Different Species

**DOI:** 10.3389/fpls.2016.02000

**Published:** 2017-01-05

**Authors:** Wei Jiang, Chengfeng Zhou, Guangting Han, Brian Via, Tammy Swain, Zhaofei Fan, Shaoyang Liu

**Affiliations:** ^1^Laboratory of New Fiber Materials and Modern Textile, The Growing Base for State Key Laboratory, Qingdao UniversityQingdao, China; ^2^College of Textiles, Qingdao UniversityQingdao, China; ^3^Forest Products Development Center, Auburn UniversityAuburn, AL, USA; ^4^School of Forestry and Wildlife, Auburn UniversityAuburn, AL, USA; ^5^Institute for Commercial Forestry ResearchScottsville, South Africa; ^6^Department of Chemistry and Physics, Troy UniversityTroy, AL, USA

**Keywords:** accurate, classification, fibrous material, identification, quantitative analysis, near infrared

## Abstract

Plant fibrous material is a good resource in textile and other industries. Normally, several kinds of plant fibrous materials used in one process are needed to be identified and characterized in advance. It is easy to identify them when they are in raw condition. However, most of the materials are semi products which are ground, rotted or pre-hydrolyzed. To classify these samples which include different species with high accuracy is a big challenge. In this research, both qualitative and quantitative analysis methods were chosen to classify six different species of samples, including softwood, hardwood, bast, and aquatic plant. Soft Independent Modeling of Class Analogy (SIMCA) and partial least squares (PLS) were used. The algorithm to classify different species of samples using PLS was created independently in this research. Results found that the six species can be successfully classified using SIMCA and PLS methods, and these two methods show similar results. The identification rates of kenaf, ramie and pine are 100%, and the identification rates of lotus, eucalyptus and tallow are higher than 94%. It is also found that spectra loadings can help pick up best wavenumber ranges for constructing the NIR model. Inter material distance can show how close between two species. Scores graph is helpful to choose the principal components numbers during the model construction.

## Introduction

Plant fibrous material is one of the most valuable materials because of its renewability, abundance and wide application (Cheng, 2009). It can be used in textile (Costa et al., 2013), paper (Hubbell and Ragauskas, 2010), food (Muangrat et al., 2010), medical (Pomin and Mourão, 2008), composite (Messing and Oppermann, 1979), biofuel (Guazzotti et al., 2003), and other areas. In each area the use of plant fibrous material is not limited to one species. Several species are normally used for one production process to ensure enough resource and yield of the product. However, different species of biomass have various properties. Therefore, identification and determination of the properties of plant fibrous material prior to process is of great significance for industrial utilization to ensure the quality of the final product.

It is easy to identify different plant fibrous materials when they are in raw condition, because they have special color, shape and structure. However, most of the materials before processing are semi products which are ground, rotted or pre-hydrolyzed (Zheng et al., 2001; Cheng, 2009). Under these conditions, the materials from different species can hardly be identified. Traditionally, they are all considered as raw material and process wet chemistry methods was used to characterize their chemical composition as guidance for the following procedure. However, wet chemistry is known to be time consuming, high pollution and complex procedure, which is not encouraged for the future (Jiang et al., 2010).

Even though the classification/identification method on plant fibrous materials have not been studied wildly, near infrared (NIR) is found to be a rapid quantitative determination method on plant fibrous material in recent years (Kelley et al., 2004; Jiang et al., 2014; Zhou et al., 2015). However, most of the NIR researches are focused on one species or several similar species (Yeh et al., 2004; Cozzolino et al., 2006; Jin and Chen, 2007; Xu et al., 2015). The limited number of work including multiple species model construction all had high prediction errors (Table 1) (Ono et al., 2003; Kelley et al., 2004; Yeh et al., 2004; Jin and Chen, 2007; Yao et al., 2010). This indicates that NIR is a good tool to fast evaluate biomass properties on either broad range with high prediction error or small range with more accuracy. A NIR modeling method which can combine broad range of species and prediction accuracy still need to be studied further.

**Table 1:** **A comparison of NIR model prediction of lignin between different species**.

**Author and year**	**Sample**	**Range of lignin content (%)**	***R*^2^**	**RMSEP (%)**	**RPD**
Jiang et al., 2014	Pine	5.45–28.59	0.99	0.6	14.34
Yao et al., 2010	*Acacia* spp.	17.9–24.9	0.94	0.53	3.01
Jin and Chen, 2007	Rice straw	7.2–12.8	0.86	2.1	0.76
Kelley et al., 2004	Agricultural fibers	0.2–35.2	0.85	5.5	1.61
Yeh et al., 2004	*Pinus taeda*	8–42	0.99	1.05	N/A
Ono et al., 2003	Forest floor	5.6–48.1	0.91	5	2.1

Some researchers found that NIR has potential ability to classify/identify samples from different species, although these researches mostly focused on food science (Barbin et al., 2012; Chen et al., 2012; Zhang et al., 2014). It is believed that high classification accuracy is much easier to achieve than quantitative analysis. If the classification model can approach 100% accuracy or close, it is easy to analyze the unknown sample's property by using a two-step prediction method. This method can first identify the species of the unknown sample, and then quantify the sample using the prediction model constructed on the corresponding species. Therefore, the NIR method of classifying/identifying plant fibrous materials is essential and worth to be studied. It is not only to classify unknown samples for pretreatment, but also a big premise for high precise quantitative analysis.

This research tried to construct an accurate classification model using NIR on six different species which were pre-ground. Soft Independent Modeling of Class Analogy (SIMCA) and partial least squares (PLS) were used to build the models, respectively.

## Materials and methods

### Sample preparation

Six species of biomass were used in this research. Southern pine (25 samples) and Tallow (24 samples) samples were harvested in Alabama, USA. Eucalyptus samples (50 samples) were shipped from South Africa. Kenaf (13 samples), Ramie (10 samples) and Lotus (17 samples) samples were collected from Xinjiang Province, Hu Nan Province and Shandong Province, respectively, in China. All the samples were ground to 40 mesh powders, and then air dried under ambient conditions. In this research, 20 southern pine samples, 20 Tallow samples, 35 Eucalyptus samples, 10 Kenaf samples, 8 Ramie samples and 14 Lotus samples were used for constructing the model. All the rest of the samples were used to verify the model accuracy.

The six species belong to three different groups. Pine is a softwood, Eucalyptus and Tallow are hardwoods. Ramie and Kenaf are bast samples. Lotus belongs to aquatic plant. These three big groups with six small species cover most of the bio-based material used in the world. The successful classification of them is very important and significant.

### Near infrared spectra collection

The NIR spectra were collected using a PerkinElmer spectrum 400 FT-IR/FT-NIR spectrometer. Biomass powders were analyzed and the reflectance spectra were collected. The spectrum covers a range of 10,000–4000 cm^−1^ with a spectral resolution of 4 cm^−1^. Each spectrum is an average of 32 scans.

### Classification method

The classification models were conducted with two different methods. One was Soft Independent Modeling of Class Analogy (SIMCA) method (Gemperline et al., 1989). The other one was partial least squares (PLS) modeling method. Prior to modeling, a spectral pretreatment was performed using multiple scattering correction (MSC) coupled with a first and second derivative with a Savitzky-Golay approach to decrease the noise of the spectra. The pretreatment can significantly reduce the noise including sample color, sample size unevenness and machine noise.

SIMCA is a statistical method for supervised classification of data. The samples in different species can be analyzed using principal components (PC) analysis. This method is used on classification of thermally modified wood in a previous study (Bachle et al., 2012).

PLS is traditionally a quantitative analysis method. In this study, we set up some rules that can use PLS to be applied on classification research. As described in Table 2, the samples that come from different species were assigned to different values (1, 2, 3…*n*). Then a PLS model was constructed based on these values. If the predicted value of the sample was inside the 0.5 error area (±0.5) of one number, this sample was identified to the relevant species.

**Table 2:** **Algorithm for classify different species samples using PLS**.

**Sample**	**Sample 1**	**Sample 2**	**Sample 3**	**…**	**Sample *n***
Sample size	*N_1_*	*N_2_*	*N_3_*	…	*N_*n*_*
Assigned value	1	2	3	…	*n*
Classification value	0.5–1.5	1.51–2.5	2.51–3.5	…	(*n* − 0.5)–(n + 0.5)
Prediction value	*A_1_–A_*N*1_*	*B_1_–B_*N*2_*	*C_1_–C_*N*3_*	…	*Z_1_–Z_*NN*_*
Recognition no.	*Nrg* = The number of sample that prediction value inside the classification value				
Recognition rate	*Nrg/N_*x*_* × 100% (*x* = 1, 2, 3,…, *n*)				
Rejection no.	*Nrj* = The number of sample that prediction value outside the classification value				
Rejection rate	*Nrj/(N_1_* + *N_2_* + *N_3_* +*…*+ *N_n_–N_x_*) × 100%				

In this research, the values of the six species were assigned as following: 1: Tallow, 2: Eucalyptus, 3: Pine, 4: Kenaf, 5: Ramie, 6: Lotus (Roughly based on the cellulose content from low to high).

## Results

### NIR spectra of all samples

**Figure 1:**
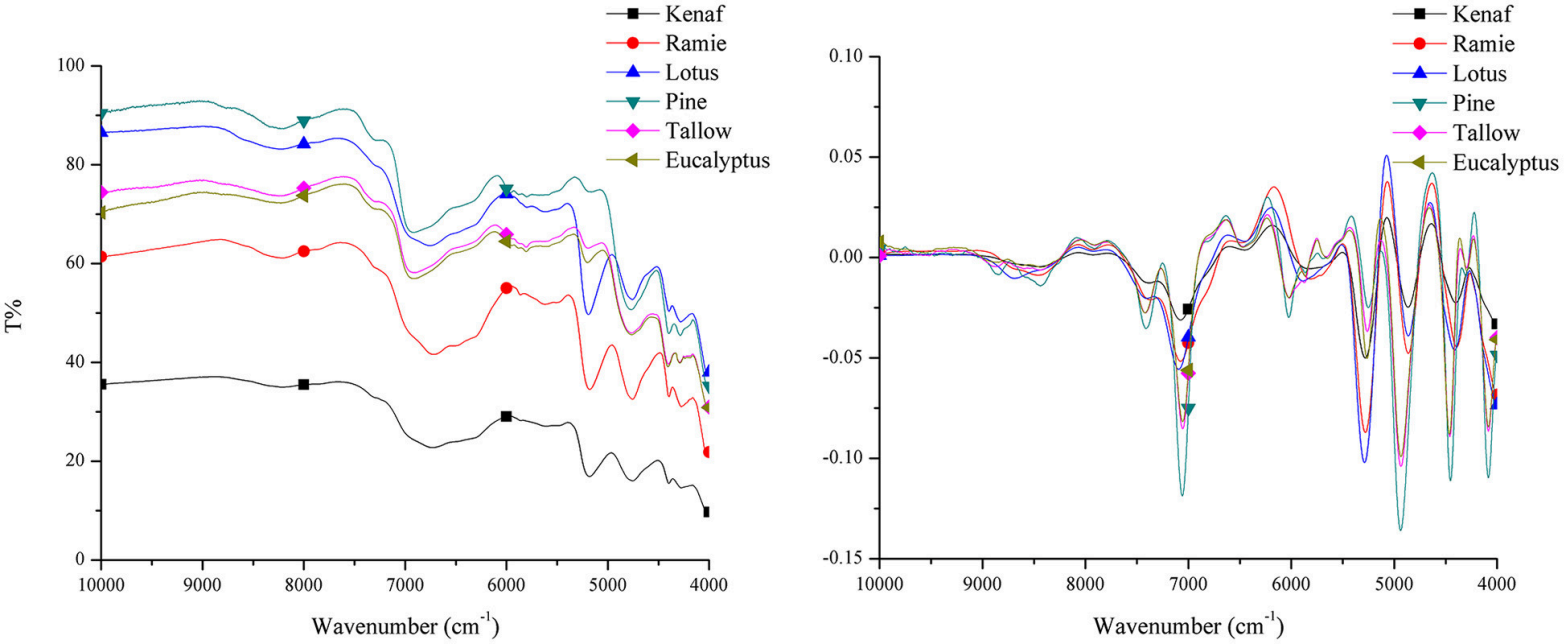
**Raw spectra (left) and First derivative spectra (right) of 6 species samples**.

By reviewing the NIR spectra of the six species in Figure 1, it is found that the six species can be clearly separated to two different groups. The wood samples including Eucalyptus, Tallow and Pine have similar spectra while Lotus, Kenaf, and Ramie hold close patterns, especially in the wavenumber range of 7500–6000 cm^−1^. This indicates that the wood samples and non-wood samples can be easily separated.

### SIMCA classification

An optimized classification model was successfully constructed using SIMCA method. It is found that the model has perfect prediction ability on Kenaf, Lotus, Ramie, Pine, and Eucalyptus (Table 3). They show 100% recognition rate and rejection rate. Tallow has 100% recognition rate while 94% rejection rate, which means the model may identify some other samples to Tallow. The identification results (Table 4) show that most of the samples were successfully identified to the correct species including Tallow. Only one Lotus samples was misidentified to other samples. As described in the previous section, Lotus is the Aquatic plant which differs from wood and bast samples; and moreover, the sample size of Lotus is not large enough. Only 14 Lotus samples were involved for the model construction and three for identification, which causes the Lotus samples not to be identified completely. In the future study, by adding more samples for model construction could help improve the accuracy at lotus species.

**Table 3:** **Classification performance report using SIMCA method**.

**Material**	**Kenaf**	**Lotus**	**Ramie**	**Pine**	**Eucalyptus**	**Tallow**
Recognition rate (%)	100 (10/10)	100 (13/13)	100 (8/8)	100 (20/20)	100 (35/35)	100 (20/20)
Rejection rate (%)	100 (96/96)	100 (93/93)	100 (98/98)	100 (86/86)	100 (71/71)	94 (81/86)

**Table 4:** **Identification result of SIMCA model**.

**No**.	**Sample ID**	**Specified material**	**Identified material**	**Result**	**Specified material total distance ratio**	**Specified material distance ratio limit**
1	Kenaf 1	Kenaf	Kenaf	Passed	0.5521	1.0000
2	Kenaf 2	Kenaf	Kenaf	Passed	0.5163	1.0000
3	Kenaf 3	Kenaf	Kenaf	Passed	0.9399	1.0000
4	Lotus 1	Lotus	Lotus	Passed	0.6424	1.0000
5	Lotus 2	Lotus	Lotus	Passed	0.8578	1.0000
6	Lotus 3	Lotus	Other	Failed	2.1082	1.0000
7	Ramie 1	Ramie	Ramie	Passed	0.6166	1.0000
8	Ramie 2	Ramie	Ramie	Passed	0.7800	1.0000
9	Pine 1	Pine	Pine	Passed	0.7980	1.0000
10	Pine 2	Pine	Pine	Passed	0.8076	1.0000
11	Pine 3	Pine	Pine	Passed	0.7657	1.0000
12	Pine 4	Pine	Pine	Passed	0.8862	1.0000
13	Pine 5	Pine	Pine	Passed	0.8500	1.0000
14	Tallow 1	Tallow	Tallow	Passed	0.7747	1.0000
15	Tallow 2	Tallow	Tallow	Passed	0.9458	1.0000
16	Tallow 3	Tallow	Tallow	Passed	0.9630	1.0000
17	Tallow 4	Tallow	Tallow	Passed	0.8836	1.0000
18	Eucalyptus 1	Eucalyptus	Eucalyptus	Passed	0.6895	1.0000
19	Eucalyptus 2	Eucalyptus	Eucalyptus	Passed	0.8127	1.0000
20	Eucalyptus 3	Eucalyptus	Eucalyptus	Passed	0.8375	1.0000
21	Eucalyptus 4	Eucalyptus	Eucalyptus	Passed	0.8184	1.0000
22	Eucalyptus 5	Eucalyptus	Eucalyptus	Passed	0.7195	1.0000
23	Eucalyptus 6	Eucalyptus	Eucalyptus	Passed	0.8553	1.0000
24	Eucalyptus 7	Eucalyptus	Eucalyptus	Passed	0.8795	1.0000
25	Eucalyptus 8	Eucalyptus	Eucalyptus	Passed	0.7072	1.0000
26	Eucalyptus 9	Eucalyptus	Eucalyptus	Passed	0.7713	1.0000
27	Eucalyptus 10	Eucalyptus	Eucalyptus	Passed	0.8578	1.0000
28	Eucalyptus 11	Eucalyptus	Eucalyptus	Passed	0.9224	1.0000
29	Eucalyptus 12	Eucalyptus	Eucalyptus	Passed	0.8840	1.0000
30	Eucalyptus 13	Eucalyptus	Eucalyptus	Passed	0.7980	1.0000
31	Eucalyptus 14	Eucalyptus	Eucalyptus	Passed	0.6793	1.0000
32	Eucalyptus 15	Eucalyptus	Eucalyptus	Passed	0.9218	1.0000

### PLS classification

**Figure 2:**
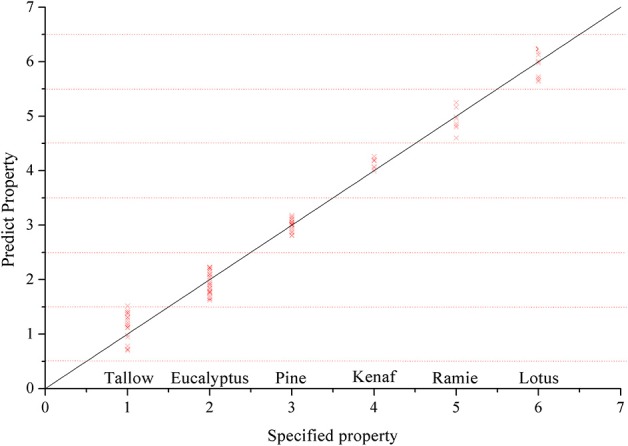
**Cross validation results using PLS**.

Another classification model was successfully constructed using PLS method with optimized parameters. The cross validation report (*R*^2^ = 98.49) shows the species have strong relevance with the number that set in previous section. The classification results were calculated based on the method of Table 2. It is found that the classification results (Table 5 and Figure 2) perfectly matched the SIMCA model, in which the Pine, Kenaf, Ramie and Lotus have excellent classification results, while Tallow and Eucalyptus slightly overlap on data.

**Table 5:** **Classification results using PLS (cross validation)**.

**Sample**	**Tallow**	**Eucalyptus**	**Pine**	**Kenaf**	**Ramie**	**Lotus**
Sample no.	20	35	20	10	8	13
Classification value	0.5–1.5	1.51–2.5	2.51–3.5	3.51–4.5	4.51–5.5	5.51–6.5
Prediction value	0.70–1.52	1.62–2.23	2.81–3.18	3.99–4.26	4.60–5.25	5.64–6.24
Recognition no.	19	35	20	10	8	13
Recognition rate	95%	100%	100%	100%	100%	100%
Rejection no.	86/86	70/71	86/86	96/96	98/98	93/93
Rejection rate	100%	98.6%	100%	100%	100%	100%

## Discussion

### Wavenumber range selection for improving classification precision

**Figure 3:**
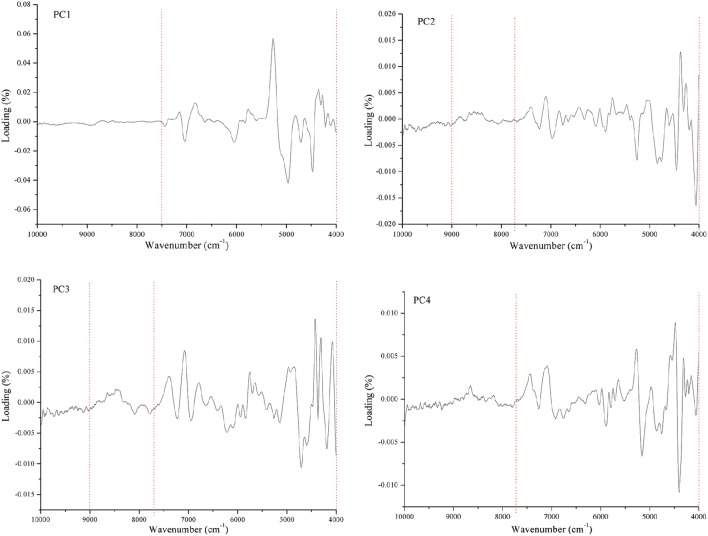
**Spectra loading plots of PC1–4 using PLS**.

**Figure 4:**
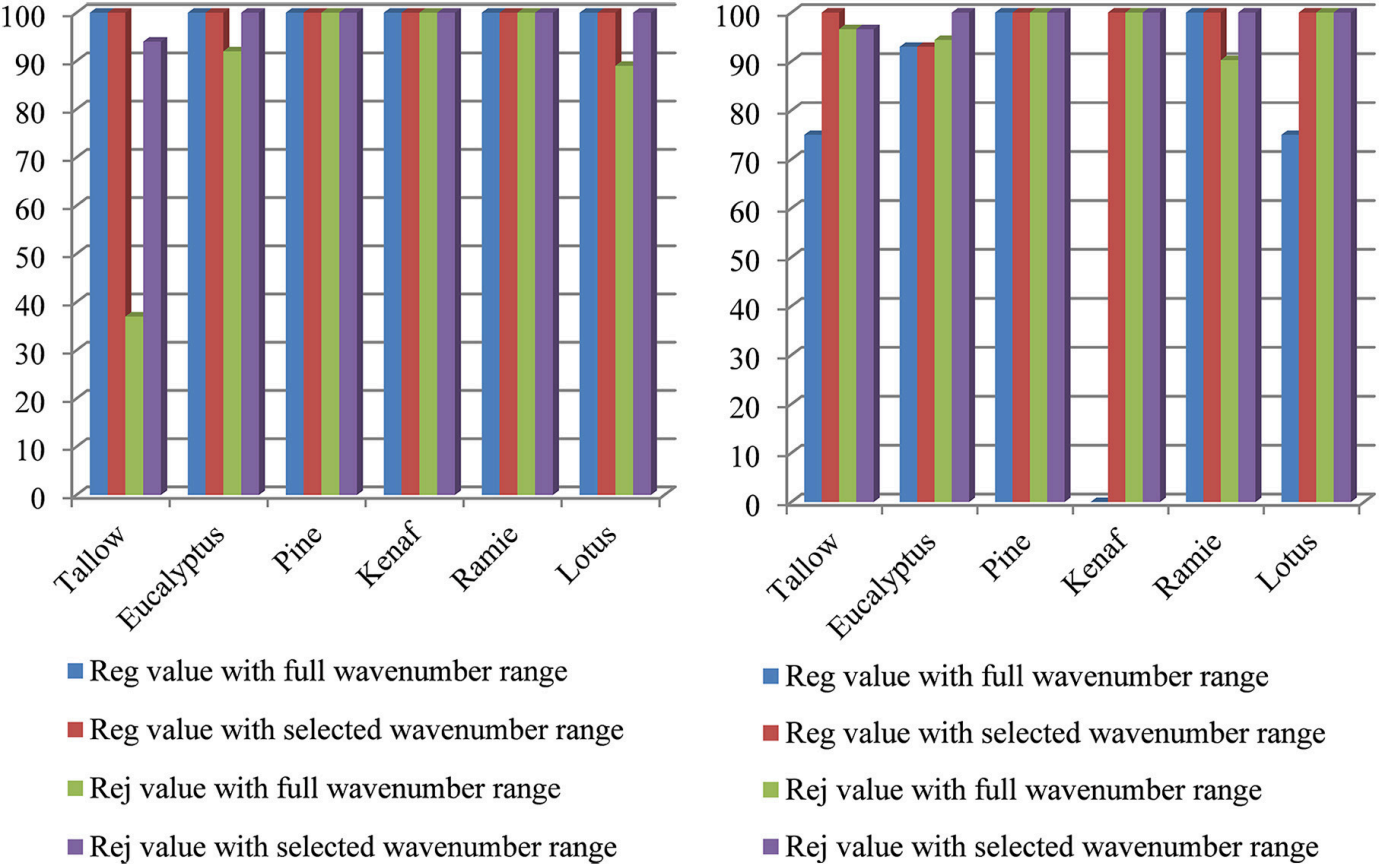
**Classification results using different wavenumber ranges for SIMCA (left) and PLS (right) model**.

**Figure 5:**
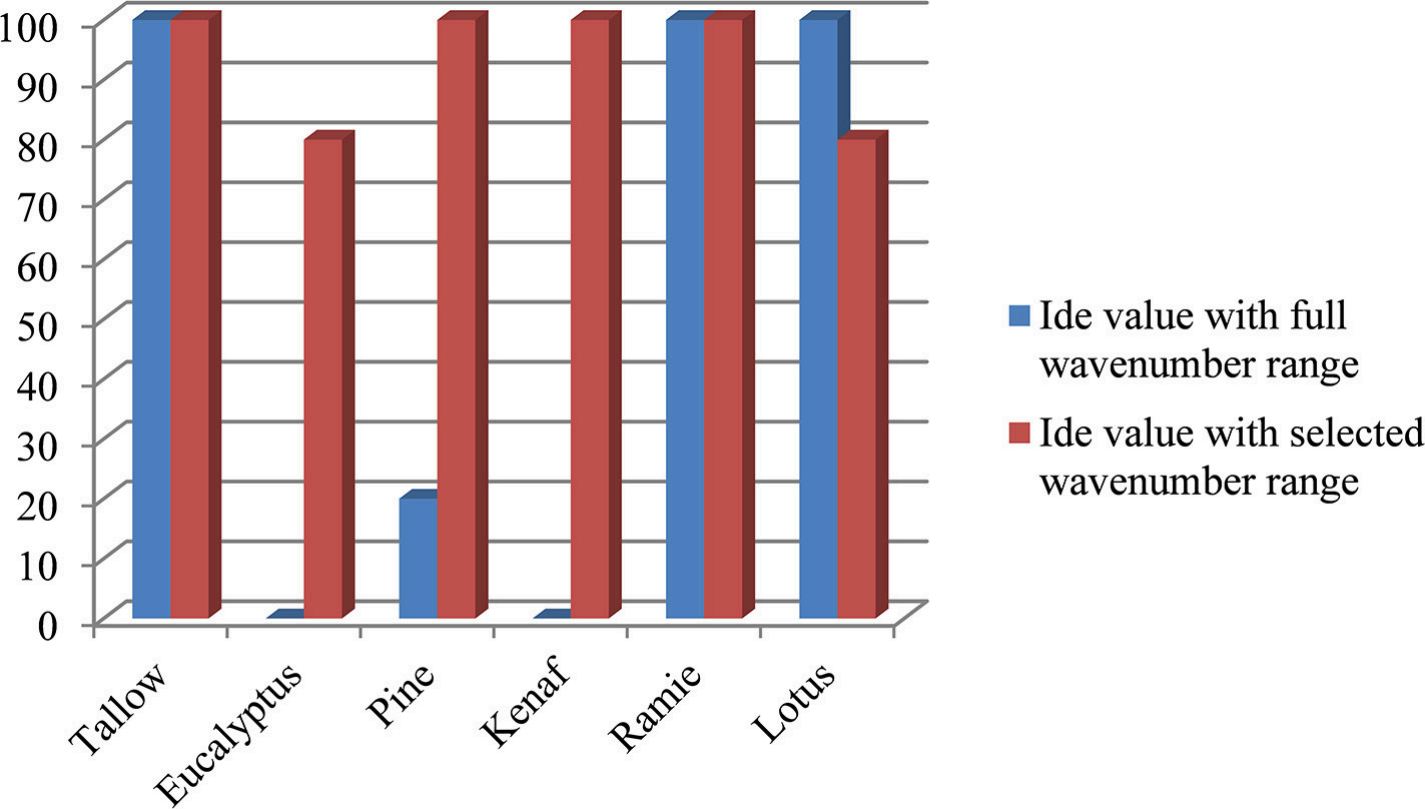
**Identification results using different wavenumber range for SIMCA model**.

This section explains how the optimized wavenumber ranges were chosen. Spectra loading plots are the data that were generated from PLS method. They show the most important information that was used in constructing the model. Figure 3 shows the spectra loading plots of PC1–4. It is found that the wavenumbers higher than 9000 cm^−1^ barely contain any useful information. The best wavenumber ranges were 7500–4000 cm^−1^ for PC 1; 7800–4000 cm^−1^ for PC2, PC3, and PC4. It is also found that 9000–7800 cm^−1^ may contain helpful information from loading plots of PC2 and PC3. Based on the above results, the wavenumber ranges of 7500–4000 cm^−1^ or (9000–7800)–4000 cm^−1^ were chosen to construct the model. It was found that the optimized wavenumber ranges are 7500–4000 cm^−1^ for SIMCA method, and 8500–4000 cm^−1^ for PLS method, respectively. Figures 4, 5 approve the above optimization. It was found that all the classification and identification performances were significantly improved by using the optimized wavenumber ranges.

### Relationship between species on classification

The study found that the Eucalyptus and Tallow samples were not perfectly classified in previous results. This section explains why this happens and how to separate them better.

Table 6 gives the inter material distance (IMD) between species using SIMCA method. The IMD shows the relationship between species: when the two species have closer relationship, the IMD will be smaller; and when the two species have big difference, the IMD will be larger. It was found that the IMDs between wood species (Eucalyptus, Tallow and Pine) and Bast species (Kenaf and Ramie) are all higher than 10, which means the wood species and bast species can be separated effortlessly. The IMDs between Lotus and Bast species and those between Lotus and Wood species are 6–10, implying that Lotus samples can be easily separated from other species. The IMD between the bast fibers (Kenaf and Ramie) is 4.69, which is lower than 6. The IMDs are all lower than 6 within wood species, the IMD between Eucalyptus and Pine is 5.29, and the IMD between Tallow and Pine is 3.8, the IMD between Tallow and Eucalyptus is the lowest value of 2.61, which can explain why the Eucalyptus and Tallow samples overlap a little during classification.

**Table 6:** **Inter material distance of SIMCA model**.

**Material**	**Kenaf**	**Lotus**	**Ramie**	**Pine**	**Eucalyptus**	**Tallow**
Kenaf	–	8.37	4.69	11.8	11.7	9.13
Lotus	–	–	9.27	12.3	11.1	8.74
Ramie	–	–	–	12.5	13.7	10.9
Pine	–	–	–	–	5.29	3.8
Eucalyptus	–	–	–	–	–	2.61

**Figure 6:**
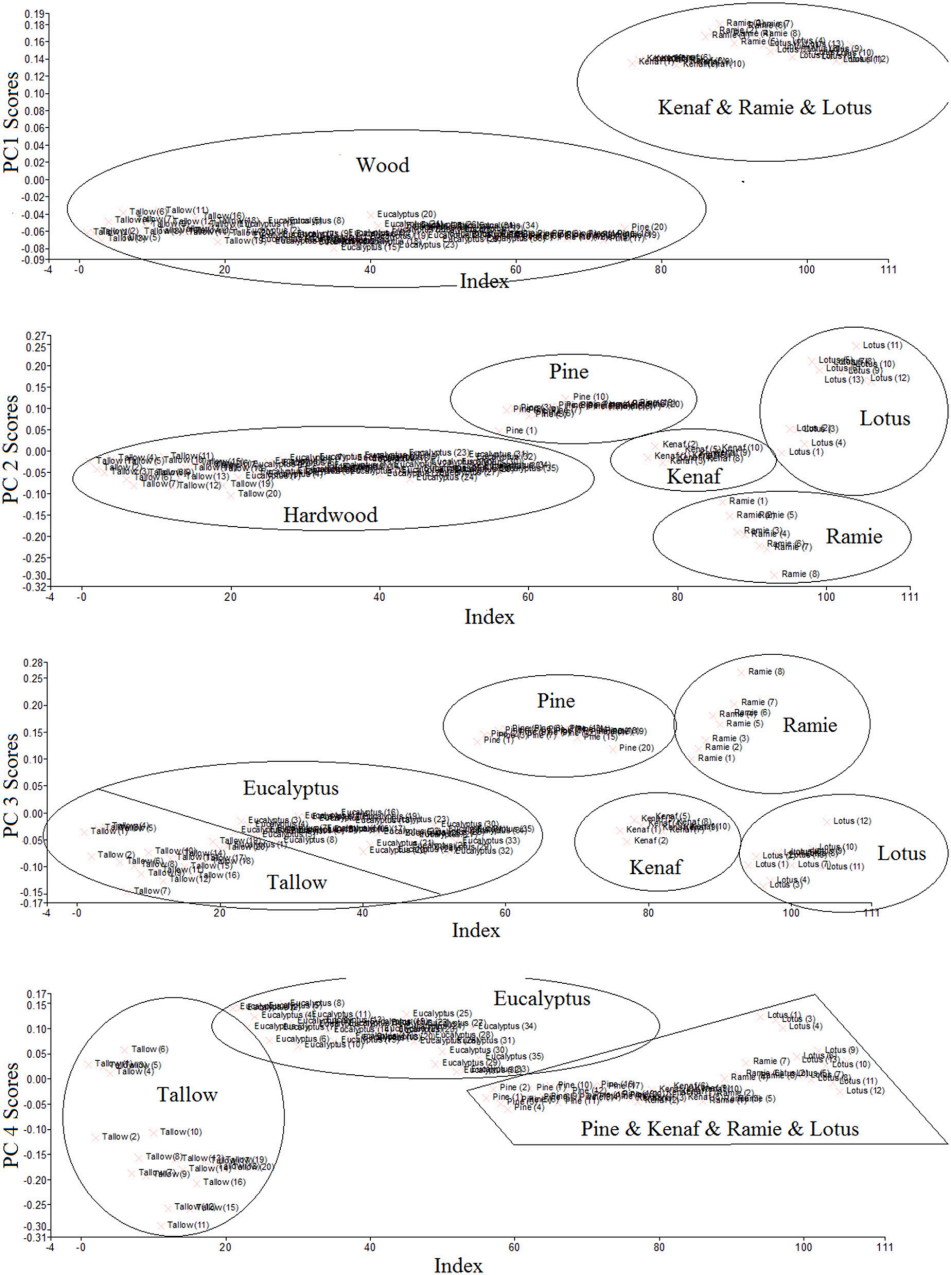
**Scores values of PC1–4 using PLS**.

Figure 6 gives the score values of all the samples for PC1–4 using PLS method. The score values show clearly how close the species are, and give us the idea on which PC we can chose to classify the species better. It was found that only wood samples (Eucalyptus, Tallow, and Pine) and non-wood samples (Kenaf, Ramie and Lotus) can be separated using PC 1. By choosing PC 2, the pine samples were separated from Eucalyptus and Tallow; Kenaf, Ramie and Lotus samples were also separated well. Eucalyptus and Tallow samples started to separate by choosing PC 3. Eucalyptus and Tallow samples were well separated when PC 4 was chosen. However, the other samples were mixed again. When choosing PC 5 (data not shown), it was found that all the samples were mixed. The data above demonstrates that combining PC1–4 are the best for classifying all the samples.

## Conclusions

The spectra of six different species samples, including Tallow, Eucalyptus, Pine, Ramie, Kenaf and Lotus, were collected and analyzed using NIR classification software (SIMCA). A new algorithm was also created to classify the six species using quantitative analysis method (PLS). Results found that the six species can successfully be classified using SIMCA and PLS methods. These two methods show similar results. The identification rete and rejection rate for all the samples were above 94%. It was also found that spectra loadings, inter material distance and scores graph were helpful for construct the model.

In the future study, with more species added in the model, the NIR model could be able to identify most of the plant fibrous species frequently used in the industry. And combined with a quantitative analysis method on each species, a wildly applicable and high precision rapid prediction system can be established and used in the future.

## Author contributions

GH and BV developed the research hypothesis and the experiment design. WJ, TS, and ZF performed sample preparation, spectra collection and SIMCA analysis. WJ and CZ performed PLS analysis and the manuscript draft. SL revised the English and discussion. The final manuscript is the end product of joint writing efforts of all authors.

## Funding

This work was supported by the Award Funds for Outstanding Middle-Aged and Young Scientists of the Shandong Province (BS2014CL044), Taishan Scholars Construction Engineering of Shandong Province, and the Program for Scientific Research Innovation Team in the Colleges and Universities of the Shandong Province.

### Conflict of interest statement

The authors declare that the research was conducted in the absence of any commercial or financial relationships that could be construed as a potential conflict of interest.
